# The Pathobiology of Myalgic Encephalomyelitis/Chronic Fatigue Syndrome: The Case for Neuroglial Failure

**DOI:** 10.3389/fncel.2022.888232

**Published:** 2022-05-09

**Authors:** Herbert Renz-Polster, Marie-Eve Tremblay, Dorothee Bienzle, Joachim E. Fischer

**Affiliations:** ^1^Division of General Medicine, Center for Preventive Medicine and Digital Health Baden-Württemberg (CPD-BW), University Medicine Mannheim, Heidelberg University, Mannheim, Germany; ^2^Axe Neurosciences, Centre de recherche du CHU de Québec, Université Laval, Quebec, QC, Canada; ^3^Département de Médecine Moléculaire, Université Laval, Quebec, QC, Canada; ^4^Department of Neurology and Neurosurgery, McGill University, Montreal, QC, Canada; ^5^Division of Medical Sciences, University of Victoria, Victoria, BC, Canada; ^6^Center for Advanced Materials and Related Technology (CAMTEC), University of Victoria, Victoria, BC, Canada; ^7^Department of Biochemistry and Molecular Biology, Faculty of Medicine, The University of British Columbia, Vancouver, BC, Canada; ^8^Department of Pathobiology, Ontario Veterinary College, University of Guelph, Guelph, ON, Canada

**Keywords:** chronic fatigue syndrome, myalgic encephalomyelitis, neuroinflammation, glia, microglia, astrocytes, oligodendrocytes, blood brain barrier

## Abstract

Although myalgic encephalomyelitis/chronic fatigue syndrome (ME/CFS) has a specific and distinctive profile of clinical features, the disease remains an enigma because causal explanation of the pathobiological matrix is lacking. Several potential disease mechanisms have been identified, including immune abnormalities, inflammatory activation, mitochondrial alterations, endothelial and muscular disturbances, cardiovascular anomalies, and dysfunction of the peripheral and central nervous systems. Yet, it remains unclear whether and how these pathways may be related and orchestrated. Here we explore the hypothesis that a common denominator of the pathobiological processes in ME/CFS may be central nervous system dysfunction due to impaired or pathologically reactive neuroglia (astrocytes, microglia and oligodendrocytes). We will test this hypothesis by reviewing, in reference to the current literature, the two most salient and widely accepted features of ME/CFS, and by investigating how these might be linked to dysfunctional neuroglia. From this review we conclude that the multifaceted pathobiology of ME/CFS may be attributable in a unifying manner to neuroglial dysfunction. Because the two key features – post exertional malaise and decreased cerebral blood flow – are also recognized in a subset of patients with post-acute sequelae COVID, we suggest that our findings may also be pertinent to this entity.

## Introduction

Myalgic encephalomyelitis/chronic fatigue syndrome (ME/CFS) is a complex, multi-system disorder with debilitating and mostly lifelong symptoms and an estimated (pre-pandemic) prevalence of 0.2 to 0.4 %. Females are approximately three times as likely to be affected than males. The disorder can develop at any age, with two peaks of incidence, one in the late teen years and another between 30 and 40 years of age. Most commonly, the disorder develops in the aftermath of acute infections, predominantly from viruses, e.g., Epstein-Barr virus, SARS coronavirus, influenza virus, Ebola virus, enteroviruses, etc. Due to a lack of established biomarkers, the diagnosis rests on clinical criteria and the exclusion of other entities [for reviews of ME/CFS, see (Bested and Marshall, 2015; Cortes Rivera et al., 2019; Bateman et al., 2021)]. An as yet undefined proportion of persons with post-acute sequelae of COVID (PASC) is predicted to also meet the criteria of ME/CFS, which may significantly add to the global disease burden (Komaroff and Lipkin, 2021; Sukocheva et al., 2021; Wong and Weitzer, 2021; Morrow et al., 2022; von Campen et al., 2022).

Given the significance of this disorder for public health and clinical medicine, the lack of knowledge regarding the pathobiology ME/CFS is an important shortcoming. This review contributes to clarification and hypothesis-generation by analyzing and interpreting literature pertaining to the pathogenesis of ME/CFS, with a focus on the possible role of glial cell populations. Glial dysfunction has frequently been postulated as a key feature of ME/CSF (Barnden et al., 2011; Nakatomi et al., 2014; Glassford, 2017; Morris et al., 2018, 2019; Staines et al., 2018; Shan et al., 2020; Nelson T. et al., 2021; Rayhan and Baraniuk, 2021), but a concise summary and in-depth discussion is still missing.

There are several reasons why ME/CFS poses a formidable research challenge. Clinically, the syndrome presents with a mélange of mostly non-specific symptoms including unrelenting fatigue persisting over more than 6 months, exertional intolerance, sleep disturbance, abnormal function in the cognitive, emotional, speech and memory domains, hypersensitivity to light and noise, psychomotor slowness and orthostatic intolerance (Bateman et al., 2021). Obviously, some of these symptoms are indicative of central nervous system (CNS) dysfunction, while others rather point to dysfunctions in peripheral organ systems. Hence, the question of whether the pathobiological basis of ME/CFS resides in the brain or periphery – or is a systemic process involving both – remains unanswered.

On the pathobiological level, ME/CFS is no less complex. In various studies, evidence of cerebral hypoperfusion, cerebral hypertension, autonomic dysregulation, muscular, metabolic, and mitochondrial dysfunction, inflammatory stimulation, redox imbalance, immune abnormalities, small fiber neuropathy, and endothelial dysfunction have been reported, among other findings (Komaroff and Lipkin, 2021). Indeed, ME/CFS may be variably described as encephalopathy, myopathy, dysautonomia, mitochondriopathy, vasculopathy or immunopathy – posing the question how all these “pathies” fit together and which ones are upstream or downstream.

However, ME/CFS is not only complex and non-specific. The disease presents with two hallmark features – one on the clinical and one on the pathobiological level – that stand out for their characteristic and defining attributes. Both are well studied, unanimously accepted among researchers, and present in *all* ME/CFS patients (at least if diagnosed according to the now internationally accepted Canadian Consensus Criteria) (Carruthers et al., 2003). Together, these features may represent leads toward a deeper understanding of ME/CFS:

### Post-exertional Malaise

While ME/CFS patients have very different baseline levels of functionality, they all have one common clinical feature: a distinctly abnormal reaction to stressful events, termed post-exertional malaise (PEM) (Stussman et al., 2020). Post-exertional malaise is described as an exacerbation of ME/CFS symptoms, which, in the same patient, can be triggered both by physical, cognitive, and mental exertion as well as orthostatic stress and sensory overload. Each patient has an individual and disease severity-dependent threshold for the development of PEM. The exercise-triggered clinical exacerbation begins after a typical delay of at least several hours post-exercise and typically persists over several days. Critical questions to be answered when elaborating pathobiological hypotheses thus include: Why does exercise-induced exacerbation of ME/CFS symptoms consistently start with a distinctive delay and persists with a distinctive duration? Why does this appear to follow an individually calibrated threshold dynamic? Why can it be equally triggered by physical, mental or cognitive exertion, as well as by sensory overload? Such an explanation also needs to account for how mental or physical exertion can trigger a wide variety of multilevel symptoms, including cognitive dysfunction, motor slowing, disturbed sensory processing and immune stimulation.

### Cerebral Hypoperfusion

Many of the pathophysiological findings in ME/CFS are contentious because they are either not well established, poorly replicable or found only in a subset of ME/CFS patients. A few pathophysiological findings, however, stand out because they seem to be uniformly present. These findings include autonomous dysfunction, metabolic abnormalities and cerebral hypoperfusion (Komaroff, 2019). In this article, we focus on the latter, because this feature is objectively and consistently identified in ME/CFS patients (for details, see section “Abnormal CBF”) (van Campen et al., 2020). In addition, cerebral hypoperfusion has been extensively studied in relation to PEM, which infers a potential common biological basis. To be answered in this regard: What causes abnormal brain perfusion, and how does this relate to the other pathophysiological features of ME/CFS such as immune dysregulation and autonomous dysfunction?

A wide range of different pathobiological explanations has been put forth to explain ME/CFS. While most hypotheses assume an immunological basis of ME/CFS, they differ as to how the immunological dysfunction may translate into the clinical manifestations. Some of these hypotheses assume a pivotal role for metabolic or mitochondrial dysfunctions (Naviaux et al., 2016), while others follow “vascular” hypotheses implying that endothelial dysfunction and/or general vascular failure may cause inadequate perfusion of both the periphery and the brain (Wirth and Scheibenbogen, 2020).

### The Concept of “Neuroinflammation” or Central Nervous System Inflammation

Lately, the investigation of inflammatory processes in the CNS has received increased attention in ME/CFS research (Glassford, 2017; VanElzakker et al., 2019). The concept of CNS inflammation (classically termed “neuroinflammation”) is still being conceptualized in detail and commonly refers to inflammatory processes taking place in the CNS to counteract infection, eliminate cellular debris or generally protect the integrity of the CNS. As an essential component of the innate immune repertoire of the CNS, this inflammation is typically mediated by the resident immune cells of the CNS, microglia, in concert with astrocytes, microvascular endothelial cells and peripheral immune cells that can migrate into the CNS. Inflammation in the CNS sets off a well-orchestrated response, which includes the release of inflammatory mediators and activation of downstream signaling pathways that can disrupt the blood-brain barrier (BBB), thus increasing perfusion and facilitating immigration of blood immune cells. The inflammatory response can also exacerbate or induce cellular stress, mitochondrial dysfunction, myelination defects and synaptic loss [for a review, see (Yang and Zhou, 2019) and (Linnerbauer et al., 2020)].

Compared with inflammation in other organs, CNS inflammation is unique. Instead of a typical neutrophil and monocyte response, the resident immune system – mainly comprised of microglia – responds, followed by subsequent delayed recruitment of blood monocytes (Perry and Andersson, 1992). Further, because the CNS is in a confined space, any swelling associated with inflammation can easily increase tissue pressure and thus give rise to reduced perfusion, ischemia, decreased venous drainage, and, possibly, raised intracranial pressure (ICP). Thus, CNS inflammation may easily result in secondary, amplified organ dysfunction (Ransohoff et al., 2003). Finally, all processes in which neuroglia become reactive can induce a vicious circle of glial priming, i.e., the induction of hyperresponsiveness to further stimulation, resulting in a self-perpetuating cycle of CNS inflammation and/or functional incompetence. By virtue of the immune and glial cells’ ability to influence the activity of other neuroglial cells, effects thereof can be conveyed to distant parts of the brain (Norden et al., 2015).

On the functional level, CNS inflammation has been associated with cytokine-mediated sickness behavior (Dantzer, 2009), excitotoxicity (Dong et al., 2009) and dysfunctional connectivity within the brain (notably due to synaptic loss and demyelination) (Rao et al., 2012) that leads to CNS dysfunction affecting sleep, circadian rhythm, emotional processing, cognition, learning and memory, pain, and autonomous regulation.

Central nervous system (CNS) inflammation is also noteworthy for enabling or amplifying other immune processes such as autoimmunity and the induction of peripheral inflammation through brain-body immune signaling. The latter implies that CNS inflammation can, through efferent vagal signaling, elicit both local and generalized inflammatory responses (Olofsson et al., 2012; Koren et al., 2021).

Possible causative mechanisms of CNS inflammation and neuroglial reactivity include direct effects through injury or infection of the brain, reactivation of endogenous microbial reservoirs in CNS cells, autoimmune reactivity with specific neural, glial, or immune system targets, repetitive mechanical strain, cerebrovascular hypertension, cerebral hypoperfusion and/or ischemia, recognition of danger-associated molecular patterns (DAMP), vagal dysfunction, norepinephrine or angiotensin II overload, or, generally, exposure to chronic stress (VanElzakker, 2013; Verkhratsky and Parpura, 2014; Calcia et al., 2016; Yang and Zhou, 2019; Živančević et al., 2021). Also, CNS inflammation can be initiated by any disruption of the BBB – caused, for instance by peripheral microvascular dysfunction (e.g., from endothelial inflammation or abnormal coagulation) or by peripheral – acute or chronic –inflammation. In the case of BBB dysfunction, neuroglial reactivity is induced by an influx of albumin, fibrinogen, among other circulating solutes, as well as blood leukocytes and their inflammatory mediators, but possibly also blood antigens, including microbial proteins that may contain pathogen-associated molecular patterns (PAMP) (Frank et al., 2021; Huang et al., 2021; Takata et al., 2021). Finally, CNS inflammation can also directly occur in response to humoral and retrograde neural signals generated by inflammation elsewhere in the body, i.e., outside the brain or spinal cord (Poon et al., 2015; Glassford, 2017; VanElzakker et al., 2019).

As the mechanisms above show, dysfunction of CNS glia may result both from inflammatory and non-inflammatory processes. By the same token, glial involvement is not necessarily *inflammatory* but may also alter CNS function *via* non-inflammatory mechanisms, e.g., neurovascular coupling (NVC) and thus blood flow distribution, modulating synaptic functions and thus neuronal connectivity and signaling, or even, especially in the case of oligodendrocyte involvement, by altering myelination. Therefore, the assumption that any glial involvement constitutes CNS inflammation is incorrect.

### The Role of Neuroglia in Brain Function and Central Nervous System Inflammation

Neuroglia in the CNS consist of heterogeneous cell populations: microglia, astrocytes, oligodendrocytes and ependymal cells. Astrocytes are neural cells of ectodermal origin that are the predominant glial cells in the brain, whereas microglia are long-lived innate immune cells of mesodermal origin. Oligodendrocytes and their progenitor cells are mainly involved in providing axonal insulation and myelination, which proceeds throughout life (Young et al., 2013). Microglia, astrocytes, and oligodendrocytes interact both with neurons and with each other *via* signaling molecules, and form long-range networks supporting and regulating the neuronal connectome (Fields et al., 2015; Hughes, 2021). Thus, they are key elements of CNS homoeostasis and protection, and in some respects interact to a degree where they can be considered one dynamic functional unit.

**FIGURE 1:**
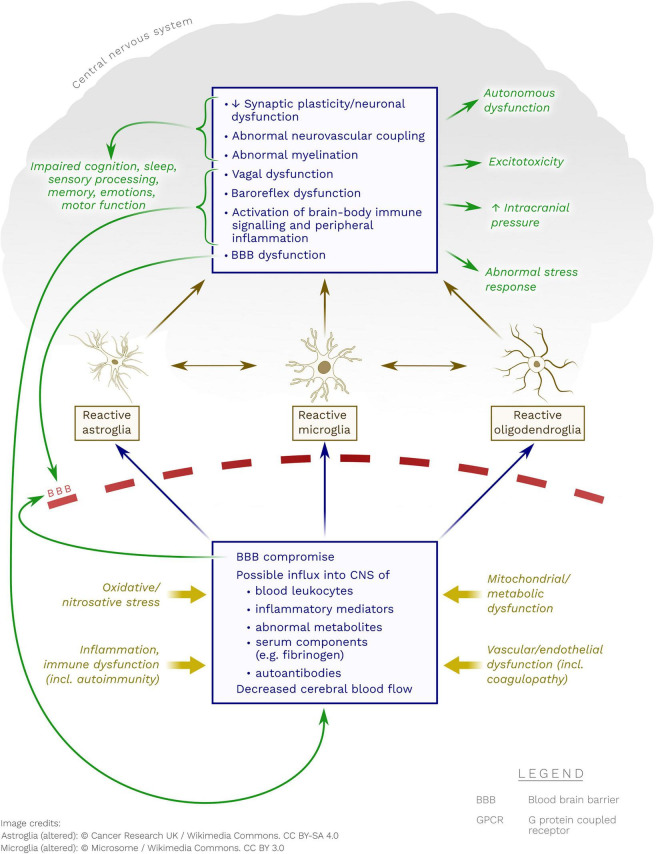
Possible pathobiological mechanisms whereby neuroglia may become reactive in ME/CFS. Note the possible contribution of immune processes (including autoimmunity to GPCR), metabolic dysfunction(s), endothelial/vascular dysfunction and oxidative/nitrosative stress.

Anatomically, neuroglial cells are most concentrated along specific white matter tracts that form functional brain units due their ability to modulate the activity of other glial cells, which also includes the possible propagation of inflammatory signals to distant locations (VanElzakker, 2013). These intrinsic brain networks reach into distant brain locations, including the limbic and prefrontal areas of the brain, which may explain why neuroglia modulate circuits involved in learning and memory (Han et al., 2012; Navarrete et al., 2012), exercise, motor function and endurance (Matsui et al., 2017; Sheikhbahaei et al., 2018). While glia heavily influence neuronal functions and the propagation of signals along neural tracts, they can also be considered an independent functional and regulatory matrix of the CNS, since they can function independently of neuronal nuclei und clusters, as well as receive input from all the major neurotransmitter systems. In addition to supporting neurons, glial cells contribute to securing adequate cerebral blood flow (CBF) by matching local blood flow to demand (NVC), and by regulating baroreflex sensitivity (Mastitskaya et al., 2020). Likewise, glial cells secure and control the BBB (Cabezas et al., 2014) and the blood-cerebrospinal fluid barrier, and thereby contribute to maintaining an adequate ICP. The glial compartment participates in modulating vagal tone and function, which is a prerequisite for an adequate stress response (Badimon et al., 2020) and for the regulation of cardiovascular, respiratory, glucoregulatory, and gastrointestinal functions (Hermann et al., 2009; MacDonald and Ellacott, 2020). At the same time, astrocytes and microglia are also essential players in the innate immune response, where they intricately interact with mast cells residing on the brain side of the BBB (Dong and Benveniste, 2001; Farina et al., 2007; Greenhalgh et al., 2020; Sofroniew, 2020). Of note, by virtue of their functional flexibility and depending on their state of reactivity, both microglia and astrocytes may have neuroprotective or pro-inflammatory properties. This milieu-dependent activity positions these neuroglial cells as a flexible link between immunity, stress response, inflammation and CNS homeostasis (see Figure 1).

### Findings Supportive of Glial Dysfunction and Central Nervous System Inflammation in Myalgic Encephalomyelitis/Chronic Fatigue Syndrome

The role of neuroglia at the intersection of immune, vascular and neuronal functions renders them attractive for focused research in ME/CFS (Noda et al., 2018). However, direct evidence is limited for CNS inflammation or neuroglial dysfunction in ME/CFS. This may be in part because neuroglial reactivity or inflammation are difficult to assess in humans due to the inaccessibility of the affected tissues for sampling. Furthermore, selective trafficking of inflammatory mediators, cellular markers, and immune cells across the BBB limit the detection of CNS inflammation based on blood or CSF analyses. Nevertheless, overexpression of CD70 on blood CD4+ T lymphocytes may identify lymphocytes with an increased potential to migrate into the CNS and thus indicate CNS inflammation (Dhaeze et al., 2019). Established and convenient blood markers for astroglial integrity like glial fibrillary acidic protein (GFAP) have not yet been investigated in ME/CFS and may not reflect neuroglial dysfunction in the absence of cellular disintegration (as seen in multiple sclerosis or traumatic brain injury). Furthermore, GFAP may also be detected in extra-neuronal tissue, which may confound results (Hainfellner et al., 2001). Other markers of glial reactivity are cytokines, complement pathway mediators, purinergic receptors, CX3C motif chemokine receptor 1 (CX3CR1), colony stimulating factor 1 receptor (CSF1R), and triggering receptor expressed on myeloid cells 2 (TREM2) (Šimončičová et al., 2021). However, these markers are largely non-specific for astrocytes and/or microglia, and can also originate from peripheral cells. In addition, their quantification in humans is not well standardized, differences between rodents and humans remain largely undefined, and specific radiotracers are not yet available. Likewise, technical challenges remain to be solved for functional CNS imaging, which may be particularly relevant to the brainstem (VanElzakker et al., 2019).

Direct evidence for a central role of neuroglial dysfunction in ME/CFS resulted from a positron emission tomography (PET) study of the translocator protein (TSPO), which labels microglia and/or astrocytes, but also endothelial and peripheral immune cells (Nakatomi et al., 2014). In this work, increased TSPO binding was observed in the cingulate cortex, hippocampus, amygdala, thalamus, midbrain, and pons of individuals with ME/CFS, and the findings correlated with the severity of neuropsychologic symptoms including fatigue sensation, cognitive impairment, pain, and depression. Indirect evidence for neuroglial involvement in ME/CFS can be gleaned from finding increased glutamate concentration in key regulatory brain areas (Gay et al., 2015; Shan et al., 2018), increased lactate in the cerebral ventricles (Mathew et al., 2009; Murrough et al., 2010; Shungu et al., 2012; Natelson et al., 2017a,b), and from magnetic resonance spectroscopy (MRS) studies showing alteration of several metabolites related to CNS inflammation and glial dysfunction (Tomoda et al., 2000; Puri et al., 2002; Chaudhuri et al., 2003; Mueller et al., 2020; Godlewska et al., 2021). Metabolites altered in ME/CFS patients include a decrease of myo-inositol, a putative marker of glial dysfunction (Noda et al., 2018), in the anterior cingulate cortex (Godlewska et al., 2021), and an increase of choline – mainly found in glial cells and thought to indicate an increased cell membrane turnover notably due to reparative gliosis – in the left anterior cingulate (Mueller et al., 2020) and basal ganglia (Chaudhuri et al., 2003). More indirect indications of glial involvement in ME/CFS have been gleaned from the consistent findings of sluggish blood oxygenation level-dependent (BOLD) signal responses to cognitive tasks (Tanaka et al., 2006; Shan et al., 2018) paradoxical activation of the default mode network (DMN) after physical exercise (Rayhan and Baraniuk, 2021), and from reduced regional fluorodeoxyglucose (FDG) uptake in the right mediofrontal cortex, brain stem (Tirelli et al., 1998) and orbitofrontal cortex (Siessmeier et al., 2003), indicating hypometabolism (Shan et al., 2020). Reduced white matter volume (Barnden et al., 2011, 2015, 2016; Finkelmeyer et al., 2018) and impaired myelination (Barnden et al., 2011, 2016, 2018; Thapaliya et al., 2020, 2021), which may indicate oligodendrocyte involvement, have been inconsistent (Shan et al., 2020). Blood and CSF analyses possibly reflective of CNS inflammation or neuroglial dysfunction in ME/CFS will be reviewed in other sections.

Thus, in summary, there are multiple paths of evidence suggestive of CNS inflammation or glial dysfunction in ME/CFS but the hypothesis remains to be proven.

## The Case for Neuroglial Dysfunction in Myalgic Encephalomyelitis/Chronic Fatigue Syndrome

To advance this quest, we have non-systematically reviewed the literature (without a specific timeframe) on the clinical and pathobiological features of ME/CFS (Renz-Polster, 2021). From the material gathered, we hypothesize that a common mechanism underlying the pathobiological basis of ME/CFS may indeed be a regulatory CNS failure due to dysfunctional or pathologically reactive neuroglia, resulting in the typical multilevel clinical manifestations of ME/CFS.

Here we test the validity of this hypothesis by an in-depth analysis of the two core features of ME/CFS to determine whether and how they might be explained by neuroglial dysfunction.

### Post-exertional Malaise

This feature of ME/CFS is noteworthy for several characteristics (VanNess et al., 2010; Arroll et al., 2014; Hartle et al., 2021):

• The clinical deterioration of PEM starts within a few hours to about a day after the triggering event, peaks in severity 2 to 3 days after exercise, and lasts from a few days to several weeks, dependent on baseline functionality and degree of exercise.

• Each ME/CFS patient seems to have an individual, severity-dependent threshold for the onset of PEM, ranging from very light (such as positional changes or even communication) to vigorous exercise or stress load.

• The exacerbation has multi-level triggers that are independent of each other. While PEM can be triggered by muscular exercise, it can, in the same patient, also be triggered by other stressors, including cognitive exercise, mental or emotional strain, orthostatic stress or sensory overload.

• Clinically, PEM is accompanied by worsening ME/CFS symptoms and loss of functional capacity (as measured with repeated cardiopulmonary exercise tests or hand grip strength). Many patients also experience symptoms of immune stimulation such as tender cervical lymph nodes and flu-like symptoms.

Several explanations for PEM have been advanced. According to one hypothesis, exercise may trigger or aggravate endothelial dysfunction thereby reducing perfusion, which may lead to muscular dysfunction as well as general, including cerebral, blood flow reduction (Wirth and Scheibenbogen, 2020). It has also been suggested that in patients with ME/CFS exercise may trigger or aggravate mitochondrial dysfunction leading to increased lactate production, reduced ATP availability in the muscles (Lien et al., 2019), abnormal levels of metabolites (Germain et al., 2022), abnormal redox balance (Paul et al., 2021) and/or nitrosative stress (Morris et al., 2017). We propose, in addition, that PEM may reflect a stress-induced aggravation of CNS inflammation or neuroglial dysfunction (which may in part be triggered by the aforementioned processes).

Glia are noteworthy for their multiplicity of phenotype, which allows a shift between “neuroprotective” and “neurotoxic” functions, depending on stimuli, physiological and pathological conditions (e.g., CNS region, stage of life, lifestyle, context of health or disease, gender) (Diniz et al., 2017, 2019; Matias et al., 2019). Upon exposure to various stimuli, microglial and astrocytes may shift to a pro-inflammatory phenotype, which increases the release of cytokines, chemokines, and neurotoxic factors thus promoting immune stimulation, CNS inflammation, and if sustained, CNS dysfunctions (Colombo and Farina, 2016; Murta et al., 2020). This reactivity can also foster a self-perpetuating hyper-response: previous encounters with inflammatory stimuli can “prime” the glial compartment for a subsequently exaggerated response. This exaggerated response can emanate from stimuli of similar (i.e., microbial antigens) or different stimuli (including inflammatory, metabolic, oxidative or nitrosative stress) (Salter and Beggs, 2014; Fleshner et al., 2017; Tay et al., 2017b; Morris et al., 2019). From a biological perspective it is therefore plausible that diverse stress signals may induce a proinflammatory state in astrocytes and microglia, which then impairs their physiological functions. Hence, stress can induce a cascade of broader CNS dysfunctions (Pearson-Leary et al., 2015; Murphy-Royal et al., 2019).

This plastic response of the glial compartment to stress signals may explain the latency aspect of PEM. The delay in onset of symptoms after exercise in ME/CFS may correspond to the time frame needed for pro-inflammatory signals to (a) reach the brain and trigger a stress response, and then (b) induce glial reactivity (to which DAMP, inflammasomes and signals from the hypothalamus-pituitary-adrenal axis could contribute) (Fleshner et al., 2017). The duration of PEM may correspond to the time for astrocytes and microglia to revert back to a more physiological state. The individual PEM threshold may reflect a variable reactivity of the neuroglial cells involved, i.e., their degree of “priming” and pre-existing impairment of their key physiological functions. The exacerbation of PEM symptoms may also reflect CNS alterations caused by the propagation of glial dysfunction into distant brain areas and regulatory centers along glial functional networks.

A possible contribution of neuroglial dysfunction to PEM can be deduced from the examination of body fluids or from CNS imaging. Our search of the literature for reported effects of exercise on blood parameters, including differential gene expressions, cytokines or complement factors, did not yield conclusive results. Pre- and post-exercise analyses of the CSF – which may be a more appropriate specimen than blood in regard to detecting abnormal processes in the CNS – have not been performed (VanElzakker et al., 2019).

For instance, while several blood analyses report altered gene expression after exercise (Whistler et al., 2005; Light et al., 2009, 2012), others do not (Keech et al., 2016; Bouquet et al., 2019; Comella et al., 2021). The same pertains to changes in cytokine or complement levels. These ambiguous results may reflect methodological differences in measuring cytokines or cytokine gene expression [reviewed in (VanElzakker et al., 2019)]. Blood cytokines originate from extravascular sites of inflammation, and their entry into circulation is hence subject to factors such as lesion perfusion, episodic release and tissue viability. These limitations may be of a magnitude that precludes robust comparison between individuals as well as between patient and control groups.

Two exercise studies based on blood samples are noteworthy since they tracked intraindividual changes or examined purified exosomes:

• In the first study, an increase of the pro-inflammatory cytokines IL-6 and IL-1β at 8 h after exercise was predictive of higher post exercise fatigue scores (White et al., 2010) (IL-6 and IL-1β trigger glial reactivity). This finding is in line with results from other fields of research which showed that, while exercise does not appear to unbalance the pro- and anti-inflammatory response in healthy people, it may lead to an unbalanced and exaggerated inflammatory response in patients with pre-existing inflammatory conditions (Cooper et al., 2007).

• The second study assessed circulating mitochondrial DNA (mtDNA) levels associated with exosomes in response to physical exercise. The exosomes from patients with and without ME/CFS were incubated with a human microglial cells and release of IL-1β was measured (Theoharides et al., 2021). The analysis showed that after exercise exosome-associated mtDNA was increased in patients with ME/CFS but not controls and that in cell culture there was significantly increased secretion of IL-1β from microglial cells.

While the study record on exercise effects in ME/CFS using blood analyses is heterogenous, the brain imaging results are more consistent. Indeed, all neuroimaging studies that have compared ME/CFS patients with healthy controls demonstrated abnormalities in response to cognitive (Barnden et al., 2015, 2016; Shan et al., 2016, 2017; Washington et al., 2020) or physical exertion (Baraniuk et al., 2021; Rayhan and Baraniuk, 2021). Some of these abnormalities were interpreted to indicate dysfunction of the glia-controlled neurovascular unit (Staines et al., 2018; Shan et al., 2020; Nelson T. et al., 2021). Most notably, in a recent BOLD functional MRI study (Rayhan and Baraniuk, 2021) of 34 ME/CFS and 24 control subjects singular effects of exercise, as not observed in any other condition, were reported. Exercise in ME/CFS patients was not only associated with a reduced global CBF but also induced increased activity in the anterior node of the DMN, a region which normally shows *decreased* activation in response to exercise. This may indicate glial dysfunction leading to disrupted coordination between functional network nodes.

How are neuroglia rendered dysfunctional by exercise? There are several potentially connected mechanisms:

#### Inflammation

Pro-inflammatory cytokines (especially IL-6) are strongly induced by exercise in healthy people (Keller et al., 2005; Light et al., 2009; Suzuki, 2019) with levels increasing up to 100-fold with maximal exertion [for a review of inflammatory effects of exercise, see Cerqueira et al. (2020) and Low et al. (2020)]. The source of the ILs released during physical exercise is thought to be mostly the muscles. The magnitude of the exercise-induced IL-6 response seems to be dependent on the intensity and especially the duration of the exercise. In high-intensity physical exercise, IL-1β is also increased. The IL response to exertion seems to be independent of the nature of the exercise (Fischer, 2006) since psychological or mental stress also induced release of cytokines (Fluge et al., 2017), including IL-6 and IL-1β, with a > 6-fold increase over baseline (Marsland et al., 2017; Moneghetti et al., 2018). Exercise-induced activation of CNS inflammation in ME/CFS appears plausible since both IL-6 and IL-1β can induce glial reactivity (Liddelow and Barres, 2017; Rosciszewski et al., 2019; Verkhratsky et al., 2019). Also, IL-6 was shown to increase mast cell proliferation and induce a more reactive mast cell phenotype (Desai et al., 2016). Similarly, and in part related to the inflammatory stimulation, exercise physiologically increases oxidative stress (Hendrix et al., 2020) and changes the metabolic matrix. In ME/CFS, where oxidative and/or metabolic regulation may be impaired (Missailidis et al., 2021; Paul et al., 2021), both processes may have separate or additive effects on glial function (see discussion).

Sympathetic stimulation, which is abnormal in ME/CFS patients at baseline (Wyller et al., 2009), may also contribute to a heightened and prolonged inflammatory milieu after exercise. Indeed, ME/CFS patients have a longer period of sympathetic stimulation after exercise (Nelson M. J. et al., 2021), which in turn may cause or aggravate several features possibly relevant to ME/CFS, including production of reactive oxygen species (ROS) (Corbi et al., 2013), endothelial dysfunction as well as immune dysfunction, notably involving B cells (Padro and Sanders, 2014; Scanzano and Cosentino, 2015). Moreover, the sympathetic hyperstimulation after exercise may, in ME/CFS patients with auto-antibodies (aAB) to vaso- and neuroregulatory G protein coupled receptors (GPCR), aggravate dysfunctional GPCR signaling and thus contribute to vascular and/or CNS dysfunctions (discussed below).

It has also been suggested (Proal and Marshall, 2018) that exercise could induce in ME/CFS patients a temporary increase in gut permeability, which in turn may induce CNS inflammation and dysfunction through the gut-brain axis (Shukla et al., 2015; Cryan et al., 2019; Carloni et al., 2021).

#### Cerebral Hypoperfusion

There is ample evidence that CBF is dysregulated in ME/CFS patients and inadequately responsive to orthostatic and cognitive challenges (for details, see section “Abnormal CBF”). This could in part contribute to PEM. Any stress, be it physical or mental, orthostatic, emotional, sensory or cognitive, places increased demands on local brain perfusion, which in ME/CFS patients may not be adequately matched with requirements due to a dysfunctional NVC. ME/CFS patients were shown to respond to exercise stimuli with hyperventilation, which in turn may add to CBF compromise (Melamed et al., 2019; van Campen et al., 2020; Natelson et al., 2022). Therefore, any allostatic load that generally or locally exceeds the regulatory capacity of the neurovascular unit may cause cerebral hypoxia and cellular energy depletion. This may directly or indirectly lead to cerebral dysfunction, e.g., by inducing reperfusion injury, lactate production, BBB disruption, cerebral edema, ROS-production, activation of the Nod-like receptor family pyrin domain-containing 3 (NLRP3)-inflammasome or other processes that induce glial reactivity and aggravate CNS inflammation (for details, see section “Abnormal CBF”).

#### Endothelial Dysfunction

Endothelial dysfunction has consistently been identified in ME/CFS (Newton et al., 2012; Scherbakov et al., 2020; Blauensteiner et al., 2021). Endothelial dysfunction may result from endothelial β2 receptor attenuation due to chronic sympathetic overstimulation or from abnormal GPCR signaling affecting vascular autoregulation (Wirth and Scheibenbogen, 2020). Many ME/CFS patients have aAB against vasoregulatory GPCR (Tanaka et al., 2003; Loebel et al., 2016; Freitag et al., 2021). Theoretically, the immune or inflammatory effects of exercise could either aggravate GPCR dysfunction directly or stimulate aAB-production and thus indirectly provoke endothelial dysfunction after exercise. Alternatively, exercise could provide a hypoxic or inflammatory milieu in which pre-existing aAB may act more effectively (Wallukat et al., 2021). It was also suggested that exercise may induce a counterregulatory release of bradykinin in the dysfunctional vasculature of ME/CFS patients (Wirth et al., 2021), which would affect permeability of the BBB (Abbott, 2000).

Any endothelial dysfunction after exercise may indeed not only affect the vascular system but also brain function through breaches in the BBB and subsequent induction of neuroglial reactivity from influx of serum components like fibrinogen, immune cells and inflammatory mediators. Fibrinogen is a key regulator of microglial reactivity (Davalos and Akassoglou, 2012), and BBB disruption associated with inflammation is now understood as a pathogenic factor in many neurological diseases (Petersen et al., 2018), and also in COVID-19 (Lee et al., 2021; Ryu et al., 2021). In the latter condition, monocyte chemoattractant protein-1 (MCP-1 = chemokine ligand 2, CCL2) was identified among the biomarkers most strongly associated with post-acute sequelae of COVID-19 (PASC) (Phetsouphanh et al., 2021) and is involved in the recruitment and transformation of microglial cells, as well as in leukocyte trafficking across the BBB (Weiss et al., 1998).

#### G Protein Coupled Receptor Dysfunction

aAB against G protein coupled receptors (GPCR) in ME/CFS patients are typically directed against angiotensin type 1 receptors (AT1R), endothelin-1 B receptors (ET1BR), and adrenergic and muscarinic acetylcholine receptors (Freitag et al., 2021), which is reminiscent of the findings in many PASC patients (Wallukat et al., 2021). In ME/CFS, the aAB levels generally correlate with disease severity (Freitag et al., 2021).

These GPCR are sometimes understood as only affecting vascular regulation. However, all the above receptors and/or their binding partners are also part of the signaling matrix of the brain and the immune system. Muscarinergic and adrenergic receptors, for instance, span a wide range of effector and regulatory functions in the brain, including memory, attention, motor control, sleep-wake-regulation and cognition (Pupo and Minneman, 2001; Scarr, 2012). Both α- and β-adrenergic receptors are most highly expressed in regions involved in autonomic activity, cardiovascular regulation and arousal (Bateman et al., 2012). α-2-adrenergic receptors (A2AR) have been shown to be important for neuroprotection (Weber et al., 2007; Gaidin et al., 2019). β-2-adrenergic receptors (B2AR) are widely expressed on glial, endothelial and immune cells, which are therefore responding to norepinephrine release (Kolmus et al., 2015). β-2-adrenergic signaling is also involved in maintaining immune tolerance (Wu et al., 2018), controlling inflammatory inputs through the vagus nerve (Vida et al., 2011) and controlling overall CNS inflammation (Junker et al., 2002; Sharma and Flood, 2018; Zhang et al., 2018). Dysfunctional β-2 signaling may therefore have substantial influence not only on autonomous regulation but also on immune responses. Similarly, muscarinic acetylcholine receptors are an intricate part of the signaling matrix of the brain. For example, the muscarinic acetylcholine receptor M3 (M3R), which may be of particular importance because of the M3R aAB found in ME/CFS patients (Loebel et al., 2016; Bynke et al., 2020), is heavily expressed in the dorsal vagal complex of the brainstem, and its stimulation on astrocytes has been related to the regulation of learning and memory (Poulin et al., 2010). The M3R is also expressed on brain microvascular endothelium (Radu et al., 2017), and M3R signaling is also important in adaptive immunity and autoimmunity (indeed, M3R is a candidate receptor for autoantigen recognition by T and B cells) (Sumida et al., 2014). This may be of particular interest given the findings of single nucleotide polymorphisms (SNPs) in genes encoding M3R in ME/CFS patients (Marshall-Gradisnik et al., 2016). Likewise, both endothelin-1 and angiotensin II and their receptors are involved in the control of immune cell migration (Cabral-Marques and Riemekasten, 2017), and ET1BR not only regulates vascular endothelial cells but also astrocytic reactivity and proliferation, and may contribute to BBB disruption and CNS inflammation (Koyama, 2021).

The effects of AT1R and MAS receptor dysfunction may extend beyond the vascular system, too. After all, activation of AT1R not only promotes endothelial dysfunction (Skultetyova et al., 2007) and vasoconstriction (possibly causing CBF reduction) but also increases peripheral and central sympathetic nervous system activity, BBB disruption (Mowry et al., 2021), oxidative stress and inflammatory activity (Wang et al., 2012), plausibly including CNS inflammation (Benicky et al., 2011). The angiotensin II/AT1R axis is an important regulatory circuit within the brain (brain-renin-angiotensin-system, b-RAS) (Bodiga and Bodiga, 2013; Cosarderelioglu et al., 2020; Nakagawa et al., 2020), which is directly and indirectly involved in baroreflex sensitivity, brain perfusion, autonomous and cardiovascular regulation, as well as vigilance, cognition and immune signaling (Wright and Harding, 2013; Abiodun and Ola, 2020; Xue et al., 2020). Indeed, dysfunctional b-RAS is now suggested to be a pathological hub in several neuropsychiatric disorders, including anxiety and depressive disorders (Labandeira-Garcia et al., 2017). Evidently, the case for a possible role of GPCR aAB within the CNS hinges on their ability to cross the BBB (see discussion).

In the clinical context, PEM presents a profoundly “inflammatory” picture, which is not only marked by an exacerbation of all baseline ME/CFS symptoms, but often goes along with signs and symptoms of general immune stimulation. This may reflect that CNS inflammation can readily induce peripheral immune responses *via* brain-peripheral body inflammatory signaling (see above). Here, a peculiar, and so far unexplained clinical phenomenon of PEM may be of special interest – the often-described tender cervical lymph nodes. We suggest that this could relate to parts of the CSF draining into cervical lymph nodes, an anatomical feature noted in rodents and ruminants (Cserr and Knopf, 1992) but not discussed with respect to ME/CFS so far. It should be considered that CNS antigens in ME/CFS patients might induce an immune responses in cervical lymph nodes *via* that route.

The above-mentioned venues through which exercise may cause clinical deterioration in ME/CFS may in part be interconnected or amplify each other. For instance, exercise could foster a pro-inflammatory milieu (if directly or through sympathetic overload), in which GPCR dysfunction may be increased, which in turn may aggravate endothelial dysfunction (or vice versa). The endothelial dysfunction may then cause or aggravate BBB dysfunction which in turn may promote neuroimmune responses, including CNS inflammation, and may also possibly allow access of GPCR to the CNS. It is also possible that exercise-induced or -aggravated mitochondrial dysfunction produces metabolic by-products that can affect glial reactivity (see discussion).

### Abnormal Cerebral Blood Flow

One of the most consistent findings in ME/CFS is abnormal global and regional CBF in response to regulatory challenges including head tilt maneuvers as well as cognitive and physical exercise (Ichise et al., 1992; Costa et al., 1995; Fischler et al., 1996; Tirelli et al., 1998; MacHale et al., 2000; Siessmeier et al., 2003; Yoshiuchi et al., 2006; Barnden et al., 2011; Biswal et al., 2011; Stewart et al., 2012; van Campen et al., 2020; Li et al., 2021; Rayhan and Baraniuk, 2021). The decrease in CBF on tilt table provocation can be independent of heart rate and blood pressure (BP) response (van Campen et al., 2020), independent of normocapnia versus hypocapnia (van Campen et al., 2021), and has also been found in the subset of ME/CFS patients with joint hypermobility (Campen et al., 2021). In these studies of CBF, the degree of global hypoperfusion correlated with symptoms and clinical severity of ME/CFS.

Indirect evidence of dynamically abnormal regional CBF comes from the consistent finding in ME/CFS patients that wider regions with greater blood oxygenation are activated in response to different tasks (Caseras et al., 2006; Mizuno et al., 2015; Shan et al., 2018). The latter has been interpreted as an indication of disrupted NVC (Staines et al., 2018; Shan et al., 2020; Nelson T. et al., 2021).

In a recent MRI study of 31 ME/CSF patients using a pseudo-continuous arterial spin labeling (PCASL) technique, significant regional CBF abnormalities in several brain regions of the limbic system were noted at rest, i.e., without orthostatic or exercise challenge (Li et al., 2021). The assumption of regional CBF abnormalities may be further supported by structural MRI findings of regional white matter loss in the left inferior fronto-occipital fasciculus (Shan et al., 2016) and in the brain stem (Barnden et al., 2011, 2015, 2016, 2018; Finkelmeyer et al., 2018), sites that are particularly sensitive to hypoxia. So far the most salient imaging evidence for abnormal CBF in ME/CFS arises from findings of a “paradoxical” reorganization of local blood flow in the anterior node of the DMN after physical exercise (Rayhan and Baraniuk, 2021).

Clearly, impaired CBF appears so central in ME/CFS that any hypothesis on the pathobiological underpinning of ME/CFS needs to account thereof. Physiologically, how well oxygen and nutrients are delivered to brain cells depends on adequate blood flow to the brain, competent vascular autoregulation, adequate NVC as well as local microvascular competence. Several hypotheses, that are not mutually exclusive, have been put forth on how the blood flow may be altered in ME/CFS.

• Abnormal CBF may be related to abnormal baroreflex function. Baroreceptors in the large blood vessels control arterial BP beat-to-beat and thus match BP with the cardiac output. If the baroreflex is not set appropriately (or if the brainstem is not interpreting the signals from the baroreceptors correctly), the cardiovascular system fails to adequately respond to the fluctuating blood flow demands upon stress (like positional changes or exercise). The presence of an abnormal function of the baroreflex in ME/CFS is supported by decreased BP variability in ME/CFS patients, indicating that they may be less able to adjust BP in response to adrenergic and vagal stimuli (Frith et al., 2012). Corroborating evidence is that CBF in ME/CFS patients can be increased by the administration of the alpha 1 agonist phenylephrine, and that cognitive dysfunction decreases with such infusion (Medow et al., 2014). Because phenylephrine does not cross the BBB, it has been speculated that the latter effect may reflect higher perfusion pressure which may overcome a hypoperfusion bottleneck in the brain, or that phenylephrine may alter the baroreceptor setpoint. Dysfunctional signaling *via* AT1R may also contribute to baroreceptor dysfunction in ME/CFS as baroreflex sensitivity is influenced by the angiotensin II/AT1R pathway (Kasparov and Paton, 1999; Gao et al., 2005; Becker et al., 2016).

• While the above may explain abnormal global CBF, it may not account for the local CBF abnormalities also typical of ME/CFS. Here, an abnormal NVC is the most likely cause, i.e., the inability of cerebral vessels to dynamically regulate blood flow in response to neural activity. This can have multiple detrimental effects including hypoxia and inadequate energy supply to activated neurons. The ensuing oxidative stress can impair endothelial cell function and thus give rise to BBB breakdown and subsequent CNS inflammation (which then may further contribute to dysregulated CBF through inappropriate vasodilation in an inflammatory milieu) (Tohidpour et al., 2017; Sankar et al., 2019).

• Blood flow in CNS tissues can also be affected by any disruption of the BBB since this allows entry of inflammatory or vasoactive mediators and thus influences microvascular function in the CNS. Indeed, through this mechanism, any form of peripheral endothelial dysfunction could translate into cerebral hypoperfusion and thus CNS dysfunction.

• Perfusion defects can also result from “mechanical” factors, and this may be relevant to the multi-etiological dimension of ME/CFS (to be discussed). Here, factors like reduced brainstem mobility (as seen in tethered cord syndrome), traumatic brain injury (TBI), repetitive strain injury [as possibly related to cranio-cervical instability/atlanto-axial instability (CCI/AAI)] or joint hypermobility syndromes may play a role. There is some evidence from research on TBI that decreased CBF may be mediated by CNS inflammation (Sankar et al., 2019).

There are several connections between CBF and the neuroglial compartment [reviewed in (Attwell et al., 2010)]. Glia have a central role both in NVC and in microvascular function. Indeed, the NVC consists of a feedforward mechanism of glutamate-driven activation of a Ca^2+^ dependent signaling pathway in both neurons and astrocytes, in response to which vasoactive factors are released to increase local blood flow (Iadecola, 2017). Also, astrocytes contribute to maintaining global CBF by virtue of their central regulatory role in the baroreflex, i.e., their ability to detect falling cerebral perfusion pressure and activate CNS autonomic sympathetic control circuits which then increase systemic arterial BP and heart rate in response (Marina et al., 2020). The baroreflex is coordinated by the nucleus of the solitary tract (NST) in the brainstem, and depends on appropriate sensing of vagal and adrenergic signals *via* NST astrocytes (Mastitskaya et al., 2020). CNS inflammatory processes involving the NST, for example, can inhibit the baroreflex centrally through ATP release from reactive NST glial cells (Mastitskaya et al., 2020). Accordingly, reactive astrogliosis in the NST has been reported after CNS trauma, infection, ischemia, stroke, and in autoimmune diseases (Sofroniew and Vinters, 2010).

Capillary-associated microglia also contribute to blood flow regulation since they are involved in regulating capillary vascular tone (Bisht et al., 2021; Kisler et al., 2021). Furthermore, similar to astrocytes, microglia contribute to the *glia limitans* (glial end-feet layer) forming the BBB around capillaries, and can structurally remodel the vasculature through the phagocytosis of endothelial cells (Haruwaka et al., 2019).

Altered CBF may have far reaching pathophysiological consequences which include many of the abnormalities observed or discussed in ME/CFS. A deficient energy supply, for instance, may induce CNS inflammation, brain edema, carotid body chemoreflex alterations and other autonomous dysfunctions, adrenergic hyperstimulation or BBB disruption. In this context, the neuroglial compartment has a potentially pivotal role. Not only is it central in the process of BBB disruption and the instigation of the inflammatory response, it also is directly affected by bioenergetic failure from hypoxia, which, among other processes, causes glutamate-induced excitotoxicity, where astrocytes play a pivotal role (Belov Kirdajova et al., 2020).

## Discussion

Despite decades of research on ME/CFS, there remains a fundamental haziness around its pathobiological matrix. The first essential debate revolves around the inception of the disease, i.e., the processes that may *initiate* ME/CFS (“How do you get ME/CFS?”). Here several hypotheses are suggested, including persistent infections, reactivation of endogenous microbial reservoirs, infection-triggered autoimmunity, or other persisting post-infectious immune dysfunctions (Komaroff and Bateman, 2021; Proal and VanElzakker, 2021).

The second debate – the focus of this paper – deals with the pathobiological pathways that may be responsible for the clinical presentation and course of ME/CFS (“What explains the symptoms of ME/CFS?”). Here, many potential contributories have been identified, including cerebral hypoperfusion, gastrointestinal dysbiosis, autonomic dysregulation, metabolic, muscular, and mitochondrial dysfunction, inflammatory stimulation, oxidative and/or nitrosative stress, immune abnormalities, autoimmunity, and endothelial dysfunction. Yet, there remains uncertainty about the sequence and direction of events, i.e., which are upstream or downstream, and which may be hubs for intersecting spokes. A similar uncertainty is common among researchers struggling to understand PASC, which in some patients is clinically indiscernible from ME/CFS (Komaroff and Bateman, 2021; Komaroff and Lipkin, 2021; Sukocheva et al., 2021; Yong and Liu, 2021).

Several “unifying” hypotheses on the most fundamental pathological underpinnings of ME/CFS have been formulated. Firstly, it has been suggested that the clinical picture of ME/CFS may reflect general cellular or metabolic dysfunction (e.g., based on mitochondrial and or peroxisomal dysfunction) (Naviaux, 2020; Missailidis et al., 2021; Che et al., 2022). These dysfunctions may cause or go along with sustained oxidative/nitrosative tissue stress (Paul et al., 2021), hypernitrosylation (Morris et al., 2017), and/or cell membrane (including channel) dysfunctions (Chaudhuri et al., 2000; Staines et al., 2018; Balinas et al., 2019). Secondly, the common pathobiological denominator underlying the clinical presentation of ME/CFS may be a general dysregulation of the vascular unit (e.g., from autoimmune processes affecting vascular receptors) (Wirth and Scheibenbogen, 2020). Thirdly, at the pathobiological core, ME/CFS may reflect regulatory CNS failure, possibly due to inflammatory changes or immune processes that may be collectively oversimplified as “neuroinflammation” (Nakatomi et al., 2014; Glassford, 2017; Mueller et al., 2020).

In this review, we probed a hypothesis related to and building upon the latter category. Based on a previous summary of the potential role of CNS glia in ME/CFS (Renz-Polster, 2021), we have analyzed the most salient and discriminating features of ME/CFS with the aim to determine if they may plausibly be explained by neuroglial dysfunction, i.e., pathological processes involving the glial cell populations, which we view as intricately linked.

**FIGURE 2:**
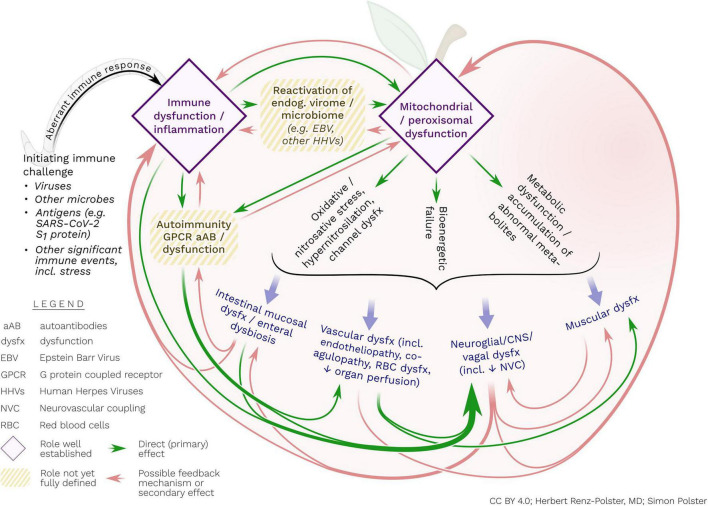
Postulated interacting pathogenic processes in ME/CFS. The disorder may ultimately represent a persistent, abnormal immune response, mostly triggered by viral and other infections. This aberrant immune response may include (or be caused by) the initiation of autoimmune reactivity (e.g., against GPCR) and/or reactivation of endogenous viruses. These – possibly interrelated – immune processes may cause concomitant mitochondrial and/or peroxisomal damage and associated metabolic dysfunctions and bioenergetic failure. The latter processes may include activated nitro-oxidative pathways, hypernitrosylation and cell membrane dysfunction, all of which may contribute to perpetuating inflammatory stimulation, endothelial and vascular dysfunction, enteral mucosal dysfunction as well as abnormal reactivity of glial cells. Glial cells may be particularly responsive to inflammatory, metabolic and oxidative/nitrosative stress. With their unique propensity for bi-directional interaction with the extra-neuronal immune system, glial cells may be the central orchestrator of the diverse disease processes of ME/CFS.

We find that altered neuroglia may indeed explain many of the core features of ME/CFS, including the multi-trigger, threshold-driven, delayed and prolonged stress response after exercise and the universal CBF deficit in response to provocative maneuvers. We therefore suggest that regulatory CNS failure due to dysfunctional or pathologically transformed neuroglia may be the central feature conveying the variable clinical presentation of ME/CFS. The processes that can ultimately trigger this glial dysfunction are multifold: a general inflammatory immune response, mitochondrial or metabolic dysfunction, autoimmune attack on GPCR, and endothelial dysfunction with subsequent breach of the BBB (see Figure 2). Our hypothesis therefore does not negate the validity of other pathobiological explanations of ME/CFS. In fact, we posit that our focus on the glial matrix of the CNS may complement other explanations by providing a more detailed understanding of the neuro-immune interface of ME/CFS. We also suggest that an exact understanding of the role of the glial cell populations may have practical bearing in regard to developing therapies for ME/CFS.

We specifically highlight that general endothelial dysfunction in the periphery – regardless of cause – is bound to affect the defensive immune system of the brain, including microglia. Likewise, we contend that immune processes in the CNS and in the periphery are inseparably linked by virtue of their vagal and humoral reciprocal communication. We therefore propose that for a better understanding of ME/CFS, micro- and macro-circulatory failure, endothelial dysfunction, metabolic dysfunction, redox imbalance, inflammatory stimulation, and CNS inflammation need to be jointly considered (see Figure 2).

### Neuroglial Dysfunction – The Link to Autoimmune Phenomena in Myalgic Encephalomyelitis/Chronic Fatigue Syndrome

We contend that our hypothesis fits with the “meta-assumption” of ME/CFS, i.e., the assumption of an underlying immune dysfunction. While the cause of the latter remains unknown, research has identified two major phenomena: autoimmunity, and evidence of a generally inflammatory milieu.

As key players in the immune responses of the CNS, both astrocytes and microglia are central to the inception of CNS autoimmunity by regulating autoantigen-presentation, BBB or blood CSF barrier breakdown and aAB leakage, as well as adaptive T- or B-cell activation for aAB production (Ikeshima-Kataoka, 2016; Baecher-Allan et al., 2018; Dong and Yong, 2019). At the same time, astrocytes and microglia closely interact with mast cells, providing another link between immune stimulation and inflammatory effects.

A subset of ME/CFS patients have abnormal levels of GPCR aAB (Tanaka et al., 2003; Wirth and Scheibenbogen, 2020; Freitag et al., 2021). It has been speculated from research on other disorders that inflammation and ischemia create an immune environment where GPCR aAB may develop, possibly establishing a vicious cycle where receptor dysfunction sustains inflammatory stimulation and/or ischemia which in turn maintains aAB production (Wallukat et al., 2021). While some authors assume that GPCR aAB in ME/CFS may primarily have direct effects on vascular regulation (Wirth and Scheibenbogen, 2020; Wallukat et al., 2021), we pointed out that GPCR aAB identified in ME/CFS also bind to immune cells and CNS targets (see section on PEM). Therefore, these aAB could also be involved in causing CNS regulatory dysfunction. Similar autoimmune processes in ME/CFS may contribute to secondary dysfunction such as small fiber neuropathy (SFN), assumed to be present in at least one third of ME/CFS patients (Grayston et al., 2019).

While the CNS is generally considered protected from peripheral aAB by an intact BBB, it may be vulnerable to autoimmune attack during states of peripheral inflammation or endothelial dysfunction that disrupt the BBB and thus allow for increased antigen presentation and immigration of immune cells (Lampron et al., 2013; Putterman, 2013). Areas of the CNS such as the circumventricular organs mediating communication between the hypothalamus and brainstem are devoid of a BBB, while dynamic immune interactions between the periphery and the CNS are increasingly described in the literature, notably upon stress and other challenges. Indeed, it has been shown that distressed glial cells can allow extravasation of peripheral immune cells, complement proteins and aAB from the bloodstream into the parenchyma of the CNS (Watkins et al., 2007), thereby allowing aAB access to neuroantigens including β2-adrenergic and muscarinic acetylcholine receptors. This process, which was shown to last for several days after initiation, is now discussed as a central pathomechanism in Complex Regional Pain Syndrome (CRPS), a disorder that clinically overlaps with ME/CFS (Cooper and Clark, 2013). It is unknown whether the GPCR aAB in the blood of ME/CFS patients can enter the CNS. While an enzyme-linked immunosorbent assay (ELISA) analysis has failed to identify GPCR aAB in the CSF of ME/CFS patients (Bynke et al., 2020), the presence of serum aAB against the muscarinic acetylcholine receptor M1 (M1R) correlated with impeded receptor binding in a brain PET study, suggesting leakage of aAB across the BBB (Yamamoto et al., 2012). Similarly, the quantity of aAB against β1 and β2 receptors in the blood correlated with brain network alterations in ME/CFS patients (Fujii et al., 2020). Suggestions for a possible role of CNS aAB have also been made for PASC (Apple et al., 2022).

The assumption of autoimmunity pertaining to regulatory neuronal structures as a causal link in ME/CFS was strengthened by experimental work on the related condition fibromyalgia. Here, the transfer of IgG from affected patients into a rodent model identified a component of the IgG fraction as symptom-inducing (Goebel et al., 2021). Apparently, this factor binds to satellite glial cells in the dorsal root ganglia, a pathomechanism that is commensurate with the clinical manifestations of the syndrome consisting of abnormalities in sensory and pain processing. Although not identified, it is plausible that this factor may be an autoantibody. This new finding from fibromyalgia research raises an intriguing question: If fibromyalgia is caused by (auto)immunity to glial cells in the spinal cord ganglia – could ME/CFS be explained by autoimmunity against glial cells in the brain?

### The Link to Other Immune Abnormalities in Myalgic Encephalomyelitis/Chronic Fatigue Syndrome

Inflammatory phenomena are unequivocally part of ME/CFS and include flu-like symptoms, tender lymph nodes or sore throat, particularly evident during disease exacerbations or PEM. Nevertheless, the immune signature of ME/CFS remains to be defined. Abnormalities in cellular immunity, including natural killer (NK) cell function, have been found, albeit inconsistently (Cliff et al., 2019; Eaton-Fitch et al., 2019). Proposed B cell abnormalities include changes in phenotype (Mensah et al., 2016), receptor repertoire skewing (Sato et al., 2021) and clonal expansion, possibly related to chronic stimulation from either microbial or auto-antigens (Milivojevic et al., 2020). It has also been suggested that the immune dysfunctions seen in ME/CFS could be a reflection of the pervasive adrenergic hyperstimulation (Scanzano and Cosentino, 2015; Nguyen et al., 2017).

Blood cytokine profiles comparing groups of ME/CFS patients with healthy controls have been contradictory, possibly owing to methodological limitation (VanElzakker et al., 2019). Relating inflammatory markers in patients to clinical symptoms has yielded some insight (Jonsjö et al., 2020). Among the cytokines correlating with symptom severity were general markers of inflammation (like IL-7, TNF-α) and cytokines that may indicate CNS-inflammation such as tumor growth factor β1 (Buckwalter and Wyss-Coray, 2004; Bureta et al., 2019), nerve growth factor (Goss et al., 1998; Linnerbauer et al., 2020), C-C motif chemokine ligand 11 [CCL11, which may influence microglial migration and reactivity (Villeda et al., 2011; Parajuli et al., 2015; Teixeira et al., 2018)], and C-X-C motif chemokine 10 (CXCL10, which was shown to mediate leukocyte influx across the BBB in a variety of inflammatory CNS diseases) (Michlmayr and McKimmie, 2014). Interestingly, the latter two cytokines were also identified in an immune network analysis of cytokines in the CSF of ME/CFS patients (Hornig et al., 2017) and as clinical markers in PASC (Fernandez-Castaneda et al., 2022). The latter study showed that even mild SARS-CoV-2 infection induced large CCL11 increases, associated with “brain fog” symptoms in the patients who developed PASC.

Findings from CSF analyses may shed a more focused light on possible CNS immune processes, and although to date only six such studies on ME/CFS patients have been reported (Lloyd et al., 1991; Baraniuk et al., 2005; Schutzer et al., 2011; Peterson et al., 2015; Hornig et al., 2017; Natelson et al., 2017a), the findings may add important information to the neuroglial hypothesis. While, similar to blood cytokine studies, comparing levels of a limited number of cytokines with healthy controls has not given a consistent picture (Lloyd et al., 1991; Natelson et al., 2005; Peterson et al., 2015), an immune network analysis in a larger sample (Hornig et al., 2017) found an altered immune signature indicative of CNS immune activation with a shift toward a T helper cell 2 (TH2, i.e., possibly autoimmune) pattern as well as increased levels of CCL11 and CXCL10, which may be indicative of CNS inflammation (Klein, 2004; Michlmayr and McKimmie, 2014; Teixeira et al., 2018). A similar network analysis of > 2500 proteins in the CSF of ME/CFS patients (Schutzer et al., 2011) identified several possibly relevant pathways, including “ß-adrenergic signaling,” “protein kinase A signaling” (role in modification of synapses and control of ion channels), “alpha-adrenergic signaling,” “GPCR signaling,” and, most significantly enriched, “CDK5 signaling.” Cyclin-dependent kinase 5 (CDK5) signaling ensures proper axonal guidance and relates to the Eph-ephrin pathway that recently emerged prominently by network analyses in the largest proteomics study in ME/CFS (to be discussed below) (Germain et al., 2021).

Basic research shows how CNS inflammatory processes can induce peripheral inflammatory processes and vice versa (VanElzakker, 2013). Key areas of this immune communication between the brain and the periphery include the neurovascular unit, the brainstem and circumventricular organs of the brain as well as the vagus nerve (DiSabato et al., 2016). A central regulatory role in this neuroimmune system is ascribed to the NST and other nuclei of the dorsal brainstem, which are not only central relay stations involved in the control of vagal input and thus of cardiovascular, respiratory, glucoregulatory, and gastrointestinal functions (Barnden et al., 2016; VanElzakker et al., 2019; MacDonald and Ellacott, 2020) but also regulate the inflammatory brain-body homeostasis (Kraynak et al., 2018). For example, when the NST detects, *via* the vagus nerve, pro-inflammatory cytokines from the periphery (such as TNF-α or IL-1β), glial cells in the NST – both microglia and astrocytes – respond by transforming their morphology and function. Their reactivity in turn contributes to eliciting an inflammatory response, involving these and other innate immune cells, in the CNS. Also, during systemic inflammation, stress-regulating brain areas are stimulated by signaling to the dorsal vagal complex within the brainstem. This mechanism is considered a physiological basis of the behavioral sickness response that humans and animals display during infections (Poon et al., 2015). It is now understood that these brain-body “mirror responses” are tightly modulated by neuroglia, both astrocytes and microglia (Godbout et al., 2005; MacDonald and Ellacott, 2020).

This central role of neuroglia as the link between central and peripheral inflammation may also apply to the gut-brain axis which has received increasing attention since intestinal microbial dysbiosis, gastrointestinal inflammation and gut barrier dysfunction are noted in ME/CFS, and may account for translocation of bacteria and dietary metabolites across mucosal barriers [for a thoughtful commentary, see (Maes et al., 2007; Sheedy et al., 2009; Frémont et al., 2013; Proal and Marshall, 2018; Komaroff and Lipkin, 2021)]. It is now not only understood that intestinal microbes are able to trigger or maintain CNS inflammation through vagal signaling, but also that microbial metabolites may have multiple roles in host physiology und influence CNS regulatory functions (Proal and Marshall, 2018). For instance, metabolites derived from dietary tryptophan crossing the BBB may affect nuclear factor κB (NF-κB) signaling [a pathway for broad innate and adaptive immune system activation (Morris and Maes, 2012)] and may phenotypically transform astrocytes and microglia (Kopitar-Jerala, 2015; Giovannoni and Quintana, 2020). Inflammatory peripheral conditions with increased intestinal permeability may induce closure of the blood-CSF barrier which in turn may negatively affect brain function by limiting the entry of nutrients and biomolecules into the CSF (Carloni et al., 2021).

### The Link to Mitochondrial and Metabolic Dysfunction

The (CNS)inflammatory milieu postulated for ME/CFS may be doubly linked to another central observation in ME/CFS, namely, metabolic, and especially mitochondrial (and possibly peroxisomal) (Che et al., 2022) dysfunction. As noted in minimal hepatic encephalopathy, even small increases in abnormal metabolites (like ammonia) can have profound effects on astrocyte and microglial function, especially in concert with inflammatory signals (Stewart and Smith, 2007; Jaeger et al., 2019; Claeys et al., 2021). It is therefore plausible that metabolic alterations and/or oxidative and nitrosative stress in ME/CFS lead to glial dysfunction (Cobb and Cole, 2015; Morris et al., 2017; Paul et al., 2021). Indeed, there is clinical overlap between ME/CFS and minimal hepatic encephalopathy. In this respect it may be notable that the first comprehensive metabolomics study following ME/CFS patients during and after maximal exercise shows an exercise-induced increase in a host of abnormal metabolites, some of which may affect or reflect pathways important for glial functioning (including glutamate dependent pathways or pathways affecting ammonia recycling) (Germain et al., 2022).

Glial reactivity or CNS inflammation could also retroact on the metabolism in ME/CFS since inflammatory stimulation or broadly restricted perfusion may cause mitochondrial dysfunction. Inflammatory activity, for instance, can cause decreased mitochondrial energy generation and mitochondrial fragmentation *via* proinflammatory cytokines and oxidative stress (Buoncervello et al., 2019; Paul et al., 2021). The latter may account for the frequent association of chronic inflammatory and autoimmune disorders with metabolic dysfunctions. Mitochondrial dysfunction may also promote oxidative stress and inflammation, which may be one of the routes through which restricted perfusion sustains inflammation (van Horssen et al., 2019), a link that apparently also pertains to CNS inflammation (Culmsee et al., 2018; van Horssen et al., 2019). Indeed, inflammatory mediators (including glutamate) produced by reactive neuroglia can trigger intracellular signaling cascades that can alter mitochondrial metabolism including respiratory chain enzyme activity (this may explain increased lactate in the CSF of ME/CFS patients) (Shungu et al., 2012).

### Not All Forms of Neuroglial Dysfunction May be “Neuroinflammation”

Does the involvement of neuroglia necessarily point to “neuroinflammation”? As we have seen, the answer is yes and no, and here we touch on the ongoing discussion about the definition of CNS inflammation and the role of glia in the CNS. Most basically, microglia can be understood as the central part of the innate immune system of the CNS. Neuroglial reactivity, therefore, may indeed be associated with or reflect inflammatory changes in the CNS or mirror inflammation outside the CNS. Inflammation constitutes defensive activity of the innate immune system, whether peripheral or central, to various types of insults including stress. This response can be beneficial or detrimental depending on the context (e.g., timing after the challenge) and magnitude, and typically involves the release of cytokines, and increased phagocytic activity. Such activity can be directed toward pathogens, but also toward stressed (yet viable) neurons and synapses (Butler et al., 2021).

At the same time, the neuroglia may be rendered reactive or dysfunctional by processes not necessarily understood as “inflammatory” – for instance through activation from abnormal vagal input or abnormal GPCR signaling (VanElzakker, 2013; Wirth and Scheibenbogen, 2020). Regardless of cause, compromised neuroglia can have a multitude of effects on the BBB, gliogenesis, neurogenesis, NVC, axonal insulation, synaptic plasticity, etc. (see Figure 1). Some hesitancy by, for instance, clinical neurologists and neuroscientists, to accept the concept of “neuroinflammation” may be resolved by a better conceptual distinction between neuroglial dysfunction and “true” CNS inflammation.

We contend, however, that there are still obstacles for clarifying the role of neuroglia in ME/CFS. First, the presence of CNS inflammation has not been unequivocally established. Second, the contributions of the different glial cell populations and their interaction remains to be defined. Third, while our hypothesis in part rests on the assumption of BBB dysregulation or disruption, this remains to be proven. Also, ME/CFS is a heterogeneous disease with varying severity and it is unknown whether the different clinical presentations share common pathobiological pathways. This is a particular challenge due to the lack of diagnostic biomarkers.

Inflammation of the CNS is not necessarily detrimental, as alluded above, since it is required for the defense against challenges and the restoration of homeostasis. A tight balance of inflammatory mediators is crucial for normal physiological processes in the CNS, including synaptic plasticity and behavioral outcomes. However, prolonged, exacerbated, or mis/dysregulated inflammation can lead to overt disease. Gaining control over the mechanisms of inflammation, but also those underlying the many physiological functions of neuroglia required to maintain health, may thus be a promising therapeutic avenue. Lastly, it should be highlighted that the neuroglial responses are highly diverse and dependent on context. Different neuroglial cell subsets and states could exert different, and even opposite functions, making it important to identify those subsets and states specifically involved in ME/CSF for the future development of therapies targeting contextually relevant functions. In this context review and further study of empirical or anecdotally successful ME/CFS therapies that target glia, such as minocycline (Plane et al., 2010; Miwa, 2021; Numata, 2021), aripiprazole (Segnitz et al., 2009; Yoneyama et al., 2014; Crosby et al., 2021), low dose naltrexone (Cabanas et al., 2021), ketogenic diet (Cossington et al., 2019), ketamine (Chang et al., 2009), vagal stimulation (Clancy et al., 2014; Meneses et al., 2016; Rodriguez et al., 2020; Namgung et al., 2022), and noninvasive transcranial neurostimulation (Gómez et al., 2021; Sabel et al., 2021; Workman et al., 2021) may be informative. Therapies that indirectly affect the innate CNS immune response, including staphylococcal vaccine (Zachrisson et al., 2002, 2004), Bacillus Calmette–Guérin (BCG) vaccine (Blok et al., 2015; Sánchez-Ramón et al., 2018), rintatolimod (Strayer et al., 2020) and stellate ganglion block should also be considered (Lipov et al., 2020; Liu and Duricka, 2022).

### Neuroglial Dysfunction May Explain Other Features of Myalgic Encephalomyelitis/Chronic Fatigue Syndrome

A central pathobiological role of neuroglia in ME/CFS may also be plausible in light of the basic functions of the cell populations involved:

• Glia are the only cells in the body that are physiologically both part of the inflammatory response AND part of the vascular-endothelial unit AND part of the functional regulatory matrix of the CNS. Indeed, neuroglia are pivotal for all functions disrupted in ME/CFS –including motor functions, autonomous regulation, sleep homeostasis, sensory gating, memory, mood and cognition.

• The involvement of glia may explain the “coupling” of clinical symptoms in ME/CFS: Mental and muscular fatigue are always occurring *concurrently*. The more a patient suffers from cognitive or mental disturbance, the less the muscles work. The more centrally fatigued a patient, the slower their gait. Central sensory dysfunction such as hypersensitivity to noise, light or touch goes hand in hand with decreased exercise capacity. Cognitive dysfunction parallels poor peripheral perfusion. Also, as disease severity increases, mental and motor dysfunctions deteriorate in unison, i.e., the less functional a patient is the more pronounced their central AND peripheral disturbances. Conversely, amelioration, temporal improvement or recovery from PEM are similarly in tandem processes. This again may be attributed to a central role of neuroglia that are *simultaneously* involved in the regulation of CNS functions, the innate immune system, the basic circuits involved in autonomous functioning, and the stress response.

• Another typical symptom in ME/CFS that may also be plausibly linked to neuroglial dysfunction is hypersensitivity to light, sound, and touch as well as sensory overload (like rapidly changing stimuli). Sensory gating in the CNS is glutamate dependent and glia play a central role. For one, astrocytes by large determine glutamate availability in the brain (Hansson and Rönnbäck, 2004; Rönnbäck and Hansson, 2004). Also, reactive microglia produce quinolinic acid (Espey et al., 1997), an excitotoxic by-product of the tryptophan-kynurenine pathway, which has been implicated in the pathogenesis of central fatigue (Yamashita, 2020) and ME/CFS in particular (Kashi et al., 2019). Indeed, increased quinolinic acid was reported in ME/CFS (Kurup and Kurup, 2003; Groven et al., 2021) and PASC (Sadlier et al., 2022). The concept of glutamate-dependent CNS excitotoxicity may explain why both cognitive impairment and sensory overstimulation occur in ME/CFS. After all, pathological activation of astrocytes can cause overstimulation of NMDA receptors, which can also affect cognitive function involving the frontal cortex (Finsterwald et al., 2015).

• Dysfunctional neuroglia may explain the neuropsychiatric components of ME/CFS. Among the most challenging aspects of ME/CFS may be anhedonia, the experience of dysphoria with admixture of anxiety, insomnia, panic, and depression, especially during exacerbations and PEM. This occurrence fits with the view that the astroglial compartment is at the center of mood regulation. Indeed, astrocytes seem to be heavily involved in the dopaminergic “reward” system (Corkrum et al., 2020). Also, in models of depression and anxiety disorders, the excitatory–inhibitory imbalance caused by astrocyte dysfunction has been implicated as an important pathogenic factor (Zhou et al., 2019). In animal models, astrocytic IL-6 mediated anxiety (Erta et al., 2015), and there is ample evidence for a role of glia, both astrocytes and microglia, in sleep regulation (Pinto et al., 2022) and neuropsychiatric disorders [for a review see (Mayegowda and Thomas, 2019)]. For details on possible cellular mechanisms see (Tay et al., 2017a; Zhou et al., 2019).

• The central role of glia may also explain the emerging recognition of mast cells in ME/CFS. Mast cells are important early effectors of the innate immune response and deeply engaged in CNS inflammation, where they may be “partners in crime” with astrocytes and microglia (Skaper et al., 2014). Central nervous system mast cells reside on the brain side of the BBB (especially in the hypothalamus, which has regions devoid of a BBB) (Rozniecki et al., 1999), and interact with astrocytes, microglia, and blood vessels (Zhang et al., 2016a; Skaper et al., 2017). *Via* their stored and newly synthesized neuroactive mediators (including IL-6 and IL-1β), mast cells can promote microglial and astrocytic reactivity (Skaper et al., 2014; Zhang et al., 2016b; Sarno, 2020) (e.g., in response to acute stress) (Huang et al., 2003), increase vascular permeability and disrupt the BBB (Theoharides and Konstantinidou, 2007).

• Finally, and possibly most importantly, astrocytes and microglia are unique among all cellular compartments in their *flexible response to stress*, which includes the formation of “stress memories”: Following pro-inflammatory stimulation glia remain in a reactive state and become hyperresponsive to subsequent stimulation because primed neuroglia respond at a different threshold than homeostatic neuroglia. Also, once repetitively stimulated by challenges, the “activation” threshold may decrease and with it the ability of the glial cells to revert to homeostatic physiological functions. In this context-dependent flexibility, neuroglia may be a prime candidate to explain the unique phenomenon of PEM (see section “Post-exertional malaise”). It may also explain why ME/CFS patients clinically deteriorate over time if they chronically exceed their “energy envelope” and why “pacing,” i.e., the strict observation of energy limits, so far remains the only effective “therapy” for ME/CFS.

### Can the Neuroglial Hypothesis Explain the Apparent Etiological Variability of Myalgic Encephalomyelitis/Chronic Fatigue Syndrome?

While ME/CFS is typically considered to be triggered by infectious agents, the disease, in a substantial subset of ME/CFS patients, is associated with connective tissue/hypermobility disorders, including the hypermobile form of Ehlers Danlos syndrome (hEDS) (Rowe et al., 1999; Barron et al., 2002; Castori et al., 2011; Bragée et al., 2020; Eccles et al., 2021). Conversely, the majority of patients with hypermobile forms of EDS meet the criteria for ME/CFS; indeed, ME/CFS is reported as ∼10 times more common in patients with EDS than in the general population (Castori et al., 2011). Neuro-orthopedic conditions of the spine and skull such as CCI/AAI, tethered cord syndrome, Arnold Chiari malformation or syringomyelia have also been reported in ME/CFS patients, and successful treatment of these conditions has sometimes resulted in recovery from ME/CFS (Brea, 2021; The Mechanical Basis of ME/CFS Craniocervical Instability, CCI, Tethered Cord, 2021).

What may render patients with hypermobile syndromes and/or these neuro-orthopedic conditions susceptible to ME/CFS? It has been suggested that connective tissue alterations may set the stage for vascular disease. Yet, in hEDS, vascular anomalies are not reported (Riley, 2020). It has been suggested that connective tissues may be damaged during the host defense against certain pathogens (Sellner et al., 2006; Singh et al., 2012), which may predispose genetically vulnerable individuals (e.g., those in the hEDS spectrum category) for subsequent over “mechanical” problems from infections.

Another lead to a possibly shared pathophysiological basis assumes that both the “hypermobile” and the “neuro-orthopedic” cases of ME/CFS impart mechanical strain on CNS tissues. In the hypermobile cases, collagen is altered in a way that allows tissue to be pulled beyond normal limits. This may interfere with the protective functions of the connective tissues and set the stage for impact injury to adjacent tissues. These strain injuries may not only result in altered joint functions (which may explain the link between hEDS and CCI/AAI) (Henderson et al., 2017) but also cause dysfunction or even injury in unprotected CNS tissue, for instance in the spinal cord, the medulla oblongata or the brainstem. Here, two of the nuclei most intensely studied in ME/CFS imaging research, the NTS and the dorsal motor complex of the vagus nerve, could be easily affected from tethering or mechanical compression and related consequences like ischemia, lactate release, hypoperfusion, edema, raised intracranial pressure, or inflammation. The same “mechanical strain” explanation may apply to neuro-orthopedic cases, as both Arnold Chiari malformation, tethered cord syndrome and syringomyelia are marked by traction on the brainstem, which may lead to chronic compression or distortion of neural and glial tissue in the brainstem or spinal cord. Pathophysiological sequelae, including reduced CBF, venous congestion and raised ICP, would have untoward effects on the functions of the neural tissues or regulatory centers. Some of these mechanical factors may be interrelated. For example, hEDS is frequently associated with occult (functional) cord tethering (Klinge et al., 2022).

The above explanations fit well with the observation that intracranial hypertension may be found in up to half of ME/CFS patients, most commonly in combination with joint hypermobility (Higgins et al., 2013; Henderson et al., 2017; Bragée et al., 2020). In an MRI study of 272 severely affected ME/CFS patients, signs of possible increased ICP were found in 83%; of these 32% were classified as definitely abnormal (Bragée et al., 2020). Signs of craniocervical obstruction were noted in 37% of patients. Several years prior, mildly increased ICP was detected by lumbar puncture in most of 20 ME/CFS patients examined; CSF drainage temporarily improved symptoms in 17 patients (Higgins et al., 2013). Interestingly, the brain area most vulnerable to alterations in CSF pressure appears to be the dorsal brainstem (Proal and VanElzakker, 2021), an area heavily implicated in studies of ME/CFS pathobiology.

It is also conceivable that the sequence of events in patients with initial “mechanical” stress includes CNS inflammation. Indeed, mouse models demonstrate how easily deformative stretch can lead to abnormal calcium influx (Wolf et al., 2001), altered gene expression (Henderson et al., 2017), proinflammatory stimulation, glial network disintegration (Koumlis et al., 2018) and apoptosis of neurons (Liu et al., 1997; Arundine et al., 2004). Activated mast cells may degranulate in response to sheer strain, tissue torsion and stretch (Hu et al., 2014; Skaper et al., 2014). The mechanical-inflammatory link is also supported by research on TBI, which was shown to be associated with CNS inflammation (and can lead to a clinical picture overlapping with ME/CFS) (Nordin et al., 2016). The CNS inflammation after TBI seems to be mediated through astrocytes and microglial-astrocyte crosstalk (Clark et al., 2019). It was also shown that spinal injuries (as to be assumed with tethered cord syndrome or syringomyelia) can induce CNS inflammation (Gwak and Hulsebosch, 2009).

### Broken Connections – May Myalgic Encephalomyelitis/Chronic Fatigue Syndrome be a Case of “Connectivity Hub Failure”?

As a speculative outlook, the role of glia in ME/CFS may even be more profound. After all, the neuroglial cell populations are now understood as part of the functional matrix of the human brain connectome (Hagmann, 2005; Sporns et al., 2005), which operates above and beyond specific brain centers, receptor units or neurotransmitter systems and integrates innate immune functions with CNS regulatory functions pertaining to autonomous regulation, sensory processing, cellular metabolism and the stress response (Fields et al., 2015; Mederos et al., 2018; Kiyoshi and Zhou, 2019).

Here, it may be informative to revisit the most remarkable characteristics of ME/CFS. This complex disease appears as a unique member of the kingdom of diseases. ME/CFS affects all functional levels of the human body, from its metazoan-like homeostatic functions such as innate immunity, inflammation, metabolism, arousal and vigilance, sleep, temperature regulation, stress response, locomotion, and digestion to the higher mammalian functions such as the reward system, mood regulation, cognition, memory and word processing. In its clinical course, the disease also occupies a special niche. ME/CFS does not follow the path of degenerative, neoplastic, chronic infectious or most autoimmune diseases that worsen over time and lead to overt tissue destruction. On the contrary, there are indications that ME/CFS – apart from complications that may arise secondarily – may be marked by the absence of long-term pathobiological sequelae. It has indeed been suggested that, if a cure for ME/CFS was found tomorrow, the vast majority of ME/CFS patients may rise out of their misery biologically unharmed (Ron Davis, in a public video) (Sheffield Me and Fibromyalgia Group, 2021). Yet, ME/CFS also does not follow the pattern of functional disorders like migraine, which are usually episodic with complete well-being in between. ME/CFS neither resolves, nor ineluctably progresses nor arises episodically from healthy states. It persists, mostly forever, with ups and downs, but within a clearly abnormal corridor.

These characteristics may indicate that ME/CFS resides at a deep *regulatory* level. This regulatory level may possibly be so profound that the question has been raised if the pathobiological circuits of ME/CFS may really be *disease* processes at all or if they may be *physiological* pathways that are normally activated only for short periods of time and under unusual circumstances (like fighting a disease or other threats) and that for some reason have become “hijacked” to remain perpetually activated (Kashi et al., 2019). Given the evidence that these pathways may include functions of the innate immune system and the stress response, we speculate that the altered pathways involve the neuroglia.

This concept may be more plausible in the light of the emerging understanding of the functional matrix of the brain [for a review, see (Stam, 2014) and (Nichols et al., 2017)]. Neuroscientists used to try to understand brain function and dysfunction starting from topographical units (brain areas or brain nuclei) to which they assigned specific functions. A similar approach was later pursued with neurotransmitters and their receptors – a certain disease, like depression, was then coined as a disease in which a certain transmitter (or receptor) system was dysfunctional.

The history of ME/CFS is marked by a similar narrative. There have been numerous hypotheses about the topographical units that is affected in ME/CFS, which variably invoked the locus coeruleus, the amygdala, the paraventricular nucleus, the suprachiasmatic nucleus or the vagal centers of the brainstem. Even a “fatigue nucleus” has been suggested as “the” area of concern in ME/CFS (Komaroff and Bateman, 2021). Likewise, several transmitter systems have been identified as dysfunctional, and variably dysfunctions in dopaminergic, serotoninergic, glutaminergic, or adrenergic transmission have been suggested as “the” biochemical basis of ME/CFS. Yet, the obvious disparity and contradictions of these topographical and biochemical explanations suggest that these may only be pieces of a broader, and probably more complex, picture.

Neuroscientists are working on a brain model to address this complexity. The human brain is now understood as a complex system with functions that depend on an optimal balance between local specialization and global integration. This framework is based on a functional matrix of interconnected pathways forming multiple large-scale functional networks (called resting-state networks – like, for instance, the DMN) (Haimovici et al., 2013; Smith et al., 2013). The analysis of the functional connectivity of these networks has identified sets of regions that can be seen as essential nodes or “hubs” for efficient neuronal signaling and communication. While these hubs are embedded in specific anatomical locations, they have functional roles across a wide range of tasks. Pathological consequences may arise from dysfunction or disconnection within or between these networks, with loss of local connectivity and short path length indicative of loss of optimal global integration (Stam, 2014). It has been suggested that such “connectivity hub overload” or “hub failure” may be a potential final common pathway of several neurological diseases, and there is now evidence of such altered functional connectivity in a wide variety of CNS diseases, including anxiety disorder, posttraumatic stress disorder, anorexia nervosa and depression (Kaplan et al., 2019). Similar changes may also occur in ME/CFS, where connectivity disturbance has been identified in the resting state and in response to tasks (Barnden et al., 2015, 2019; Gay et al., 2015; Shan et al., 2016, 2017; Rayhan and Baraniuk, 2021).

Interestingly, and in support of our general hypothesis, there is evidence that glia - themselves organized as an interdependent syncytial network (Kalafatakis and Karagogeos, 2021; Rayhan and Baraniuk, 2021) may be paramount to guarantee effective connectivity and hub integrity in the neuronal connectome, which relies on constant axonal and synaptic reorganization (Spiegler et al., 2016). By virtue of their metabolic and regulatory support for neurons as well as their role in neurite outgrowth, neuronal guidance, maintenance of the axon-myelin interface and synaptic plasticity, dysfunctional neuroglia may initiate hub failure (Fields et al., 2015; Lu et al., 2019).

A noteworthy observation is that the largest human proteomics dataset analyses of proteins unique to the ME/CFS patients robustly highlighted the Eph/ephrin pathway (Germain et al., 2021). The latter contributes to “guiding” migrating cells and has attracted attention for its unique bi-directional signaling between astrocytes and neurons, and its influence on synaptic plasticity and axon tract formation and pruning (Yang et al., 2018). Eph/ephrin appears to also regulate oligodendrocyte precursor cells and oligodendrocytes, since they contribute to axonal insulation and may be involved in extracellular glutamate homeostasis and the regulation of excitotoxicity. Interestingly, Eph receptors are highly expressed in brain regions with morphological and physiological plasticity, including the amygdala and hippocampus, areas of particular interest in ME/CFS research (Shan et al., 2020; Li et al., 2021). Proteomic analysis of CSF (Schutzer et al., 2011) and circulating EVs (Eguchi et al., 2020) also highlighted axonal guidance pathways linked to Eph/ephrin as possibly abnormal in ME/CFS. These observations fit with recent findings of peroxisomal dysfunction and associated depletion of plasmalogens in ME/CFS (Che et al., 2022), as peroxisomes are not only heavily enriched in neuroglial cells but are also paramount for the preservation of axonal integrity, the formation and maintenance of myelin, immune function and general brain health [for reviews, see (Baes and Aubourg, 2009) and (Kassmann, 2014)]. If only on a speculative note, in the largest ME/CFS genome-wide association study to date, the most significant risk loci association was found for the tubulin polymerization promoting protein (TPPP) gene region (Hajdarevic et al., 2022). Tubulin polymerization promoting protein is expressed in brain tissues and plays a central role in myelination (Fu et al., 2019).

In summary, although this review in part builds on hypotheses still to be substantiated or refuted, evidence is strong that the neuroglia, as a cellular network regulating autonomous functions, the immune system and the stress response, and that reaches across and beyond specific brain nuclei, transmitter and receptor systems, is central to the pathogenesis of ME/CFS. Considering their functional importance at the interface of central and peripheral biological functions, we propose greater focus on the role of neuroglia in ME/CFS (and PASC) research.

## Data Availability Statement

The original contributions presented in the study are included in the article/supplementary material, further inquiries can be directed to the corresponding author.

## Author Contributions

HR-P conceptualized the work and drafted the manuscript. M-ET and DB revised and expanded the concept and also reviewed, edited, and critically revised the article. All authors discussed the literature base and finally read and approved the final manuscript.

## Conflict of Interest

The authors declare that the research was conducted in the absence of any commercial or financial relationships that could be construed as a potential conflict of interest.

## Publisher’s Note

All claims expressed in this article are solely those of the authors and do not necessarily represent those of their affiliated organizations, or those of the publisher, the editors and the reviewers. Any product that may be evaluated in this article, or claim that may be made by its manufacturer, is not guaranteed or endorsed by the publisher.
